# Validating Older Adult Morbidity Trajectories Using Multiple Comorbidity Indices

**DOI:** 10.1093/geroni/igaa057.564

**Published:** 2020-12-16

**Authors:** Michael Newman, Heidi Hanson, Karen Schliep, Samir Abdelrahman, Jim VanDerslice, Ken Smith, Christy Porucznik

**Affiliations:** 1 University of Utah, Holladay, Utah, United States; 2 University of Utah, Salt Lake City, Utah, United States; 3 University of Utah, Salt Lake City, United States

## Abstract

Many older adults lead healthy lives while aging, with little or no morbidity. This group has been identified as “Escapers”, for escaping the 10 most common lethal diseases in older adults. “Morbidity Trajectories” (MOTRs) are a metric based on the temporal patterning of comorbidity, which is used to characterize changes in disease status as a person ages. While these trajectories have been used to identify Escapers in various populations, they are sensitive to the choice of the disease metric. This study seeks to describe the differences in MOTR scale by alternative comorbidity indices. Understanding these differences is important because of the need to validate the potential end-point in health trajectory risk scores that may be used in a clinical setting. We found that 15-19 percent of a Medicare utilizing population (n=321722) aged >= 65 between 1992 and 2012 fall into the Escaper category, where there is a consistent Quan modification Charlson Comorbidity Index (CCI) score of 0 during the entire study period. Using the vanWalraven (vW) Elixhauser Comorbidity Index modification, we found that about a third (35.2%) of the study population have a vW Elixhauser score of 0 over the span, a significantly higher portion than the CCI estimate. We will discuss this difference and the resulting varying trajectories from each of these indices. Future work includes further validation of the MOTR scale using unsupervised machine learning clustering methods, and using supervised machine learning models to identify clinical factors and early life conditions that may influence MOTR membership.

